# Idiopathic recurrent pericarditis in children: a tertiary care cohort highlighting the emergence and effectiveness of interleukin-1 blockade

**DOI:** 10.1007/s00431-026-07074-3

**Published:** 2026-05-15

**Authors:** Aybuke Gunalp, Nergis Akay, Merve Kirali, Zeynep Torunoglu, Ece Aslan, Umit Gul, Elif Kilic Konte, Nida Gulderen Kalay Senturk, Mehmet Yildiz, Fatih Haslak, Amra Adrovic, Sezgin Sahin, Kenan Barut, Reyhan Dedeoglu, Ozgur Kasapcopur

**Affiliations:** 1 Cerrahpasa Faculty of Medicine, Department of Pediatric Rheumatology, Istanbul University-Cerrahpasa, Istanbul, Turkey; 2 Cerrahpasa Faculty of Medicine, Department of Pediatrics, Istanbul University-Cerrahpasa, Istanbul, Turkey; 3 Cerrahpasa Faculty of Medicine, Department of Pediatric Cardiology, Istanbul University-Cerrahpasa, Istanbul, Turkey

**Keywords:** Idiopathic recurrent pericarditis, Pediatrics, Interleukin-1 inhibitors, Anakinra

## Abstract

Idiopathic recurrent pericarditis (IRP) is an increasingly recognized inflammatory condition in children, characterized by recurrent flares and therapeutic challenges, with emerging evidence supporting a role for interleukin-1 (IL-1)-mediated pathways. This study aimed to characterize the clinical features of a pediatric IRP cohort at our center, to examine clinically relevant differences between patients requiring and not requiring anakinra, and to evaluate anakinra effectiveness by assessing changes in disease activity before and after treatment. In this single-center retrospective cohort study, 26 children with IRP were evaluated in terms of demographic, clinical, laboratory, genetic, and treatment characteristics. Patients were stratified according to anakinra requirement, and these characteristics were compared between groups. In patients receiving anakinra, treatment effectiveness was assessed by comparing disease activity before and after therapy using annualized attack rate (AAR) and inflammatory markers. The cohort showed a male predominance (19/26, 73%), with a median age at diagnosis of 114 months (IQR, 48–168). Chest pain was the most common presenting symptom (16/26, 62%). Pericardial effusion was present in all patients (26/26, 100%), while pleural effusion was observed in 12/26 (46%). Despite conventional therapy with nonsteroidal anti-inflammatory drugs (NSAIDs), colchicine, and corticosteroids, steroid dependency developed in 9/22 patients (41%). Anakinra was required in 16 patients (62%) and was associated with a marked reduction in disease activity, with a median relative decrease in AAR of 89.4% (IQR, 56.7–100%); a ≥ 50% reduction in AAR was achieved in 12/16 patients (75%). Significant and concordant reductions in inflammatory markers were observed. Relapse frequently occurred during anakinra dose spacing or discontinuation, while corticosteroid withdrawal was achieved in most treated patients. Patients requiring anakinra had higher baseline disease activity. *Conclusion*: Idiopathic recurrent pericarditis in children represents a heterogeneous and frequently relapsing inflammatory condition in which conventional therapies often fail to achieve sustained disease control. As an IL-1 blockade, anakinra emerges as a key therapeutic option, enabling corticosteroid sparing while effectively achieving disease control through targeting underlying innate immunity pathways.

**Table:** 

**What is Known:** • *Idiopathic recurrent pericarditis (IRP) in children is characterized by recurrent inflammatory flares and frequent treatment resistance, with conventional therapies (NSAIDs, colchicine, and corticosteroids) often being insufficient and associated with steroid dependency.* • *Increasing evidence implicates autoinflammatory pathways in disease pathogenesis, highlighting the potential role of IL-1 inhibition in refractory cases.*
**What is New:** • *The high prevalence of MEFV-associated variants, frequent steroid dependency, and substantial use of IL-1 inhibitors collectively support an auto inflammatory-driven disease model in pediatric IRP.* • *Anakinra markedly reduces disease activity even in patients with high baseline inflammatory burden; however, sustained disease control requires continuous therapy, as dose spacing is associated with increased relapse rates.*

**What is Known:**

• *Idiopathic recurrent pericarditis (IRP) in children is characterized by recurrent inflammatory flares and frequent treatment resistance, with conventional therapies (NSAIDs, colchicine, and corticosteroids) often being insufficient and associated with steroid dependency.*

• *Increasing evidence implicates autoinflammatory pathways in disease pathogenesis, highlighting the potential role of IL-1 inhibition in refractory cases.*

**What is New:**

• *The high prevalence of MEFV-associated variants, frequent steroid dependency, and substantial use of IL-1 inhibitors collectively support an auto inflammatory-driven disease model in pediatric IRP.*

• *Anakinra markedly reduces disease activity even in patients with high baseline inflammatory burden; however, sustained disease control requires continuous therapy, as dose spacing is associated with increased relapse rates.*

## Introduction

Pericarditis is an inflammatory disorder of the pericardium and an uncommon but clinically significant cause of chest pain in children, accounting for approximately 5% of chest pain–related emergency department presentations [1]. Although acute pericarditis is often self-limited, recurrent pericarditis represents the most challenging disease phenotype, defined by repeated inflammatory relapses following a symptom-free interval of at least 4–6 weeks [2, 3]. Recurrent pericarditis develops in up to 30–35% of pediatric patients after a first episode and, in the absence of an identifiable secondary cause, is classified as idiopathic recurrent pericarditis (IRP) [1]. Compared with adults, pediatric IRP is less frequent but often more inflammatory, with higher acute-phase reactants, prominent systemic symptoms, and a greater tendency toward corticosteroid dependence, resulting in a substantial impact on quality of life and long-term outcomes [4, 5].

The pathogenesis of IRP remains incompletely understood. Historically considered an autoimmune condition based on its association with systemic autoimmune diseases and responsiveness to corticosteroids, growing evidence, particularly from pediatric cohorts, supports a major role for innate immune pathways driving autoinflammation. Abrupt febrile attacks, striking elevations of inflammatory markers, complete intercritical recovery, and clinical overlap with monogenic autoinflammatory diseases such as familial Mediterranean fever (FMF) and TNF receptor–associated periodic syndrome (TRAPS) point toward dysregulation of innate immunity [6, 7]. Central to this paradigm is the interleukin-1 (IL-1) pathway, whose pivotal role is underscored by the rapid and sustained efficacy of IL-1 blockade, especially with anakinra, in colchicine-resistant or corticosteroid-dependent IRP [8, 9].

Despite a generally favorable survival prognosis, IRP remains a therapeutic challenge in children, as recurrent inflammation and prolonged corticosteroid exposure may result in significant morbidity, while rare but serious complications such as cardiac tamponade, myocardial involvement, and constrictive pericarditis can occur. Current management strategies are largely extrapolated from adult data, despite age-related differences in immune phenotype and disease behavior [1]. Given the limited pediatric-focused evidence, this study aimed to characterize the clinical features of a pediatric IRP cohort at our center, to examine clinically relevant differences between patients requiring and not requiring anakinra, and to evaluate the effectiveness of anakinra by assessing changes in disease activity before and after treatment.

## Materials and methods

### Study design and patient selection

This study was conducted as a retrospective observational cohort study at a tertiary referral center and focused exclusively on pediatric patients with IRP. Patients were included when a first episode of pericarditis occurred between January 2013 and January 2025 and was followed by at least one recurrence, defined as the reappearance of pericardial symptoms after complete resolution of the initial episode with a symptom-free interval of at least 4–6 weeks, in the absence of an identifiable alternative etiology. Excluded alternative causes included systemic autoimmune diseases (such as systemic lupus erythematosus or systemic vasculitis), genetically confirmed monogenic autoinflammatory disorders (including FMF or TRAPS), and infective, neoplastic, metabolic, drug-related, post–cardiac injury–related pericarditis or rare genetic syndrome (including camptodactyly–arthropathy–coxa vara–pericarditis syndrome(CACP)), thereby fulfilling the criteria for idiopathic recurrent pericarditis [10–12]. All patients were followed in the pediatric rheumatology outpatient clinic at Cerrahpasa Medical Faculty for a minimum duration of 6 months. Patients with irregular follow-up or more than 50% missing data were excluded from the study.

### Data collection

Data collected for each patient included demographic characteristics (sex, age at diagnosis, and duration of follow-up) and clinical features at presentation (chest pain, dyspnea, fever, abdominal pain, pericardial effusion, and pleural effusion). Procedural history, including pericardiocentesis and pericardiectomy, was also recorded. Genetic data comprised the presence of MEFV variants and results of extended autoinflammatory gene panel testing where available. Baseline inflammatory markers, including annualized attack rate (AAR), C-reactive protein (CRP), and erythrocyte sedimentation rate (ESR), were documented. Treatment-related data included the use of nonsteroidal anti-inflammatory drugs (NSAIDs), colchicine, and systemic corticosteroids, as well as steroid response and dependency. Detailed information on anakinra therapy was collected, including time from diagnosis to initiation, dose (anakinra was administered at doses up to 5 mg/kg/day according to institutional practice, and further dose escalation was not required as adequate disease control was achieved in most patients), treatment duration, attack frequency under treatment, attempts at dose spacing, relapse after spacing, and discontinuation attempts and outcomes. Data on canakinumab therapy were also recorded. In addition, changes in CRP, ESR, and AAR before and after anakinra treatment were assessed, along with treatments at the last follow-up. All data were retrospectively reviewed from hospital records, including both paper charts and electronic medical records.

### Diagnostic, disease activity, and treatment response definitions

Initial and recurrent episodes of pericarditis were diagnosed in accordance with the European Society of Cardiology (ESC) guidelines [3, 13]. A definitive diagnosis required the presence of at least two of the following criteria: typical pericardial chest pain, a pericardial friction rub on auscultation, characteristic electrocardiographic changes including new widespread ST-segment elevation and/or PR-segment depression, and new or worsening pericardial effusion detected by transthoracic echocardiography. Recurrent pericarditis was defined as the reappearance of pericardial symptoms after resolution of an initial episode, following a symptom-free interval of at least 4–6 weeks.

Disease activity and treatment response were assessed using predefined clinical and laboratory criteria, in accordance with previously published definitions and international guideline recommendations [3, 14]. Disease activity was longitudinally assessed using the annualized attack rate (AAR) and serial acute-phase reactants (CRP, ESR). Treatment response was defined as symptomatic and laboratory improvement without rescue therapy, whereas partial response was defined as clinical improvement with persistent inflammation and/or treatment escalation. Treatment resistance was defined as persistent or recurrent disease despite adequate nonsteroidal anti-inflammatory drug and colchicine therapy (colchicine was administered according to age- and weight-based recommendations, with dose escalation in therapy-refractory cases up to a maximum of 2 mg/day). Steroid dependency was defined as relapse during corticosteroid tapering or after discontinuation. In patients receiving interleukin-1 blockade, response was additionally assessed based on symptom resolution (≥ 50% reduction in AAR from baseline), laboratory normalization, and successful steroid tapering.

To account for heterogeneity in follow-up duration across patients and to enable standardized comparisons of disease activity, attack frequency was expressed as the annualized attack rate (AAR), defined as the number of documented attacks per patient-year of follow-up. This approach reduces bias related to unequal observation periods and allows clinically meaningful assessment of within-patient change and treatment response.

### Ethical considerations

This study was approved by the Clinical Research Ethics Committee of Istanbul University–Cerrahpaşa Faculty of Medicine. Written informed consent was obtained from the legal guardians of all participants in accordance with the Declaration of Helsinki.

### Statistical analysis

All statistical analyses were performed using IBM SPSS Statistics (version 26). Normality was assessed using the Shapiro–Wilk test and visual inspection. Continuous variables were summarized as median (IQR) or mean ± SD, as appropriate. Within-patient pre- and post-treatment comparisons were performed using the Wilcoxon signed-rank test for nonnormally distributed variables, with effect sizes calculated as *r* = *Z*/√*N*. For CRP and ESR, paired analyses with bootstrap resampling were conducted and effect sizes were reported as Cohen’s dz. Between-group comparisons were performed using the Mann–Whitney *U* test for nonnormally distributed variables and Welch’s *t*-test for approximately normally distributed variables. Categorical variables were compared using the *χ*^2^ test or Fisher’s exact test, as appropriate. All tests were two-sided, and *p* < 0.05 was considered statistically significant.

## Results

### Baseline characteristics of the study cohort

A total of 26 patients were included in the study; the majority were male (*n* = 19, 73%), with a wide age range at diagnosis [median 114 months (IQR 48–168)] and a prolonged follow-up duration [median 72 months (IQR 36–96)]. Chest pain was the most common presenting symptom (*n* = 16, 62%), frequently accompanied by dyspnea and fever (*n* = 11, 42%, and *n* = 5, 19%). Pericardial effusion was observed in all patients, and a substantial proportion required pericardiocentesis (*n* = 11, 42%). Genetic analysis identified at least one genetic variant in 11 patients across the cohort. Most detected variants involved the MEFV gene, supporting the prominent role of autoinflammatory pathways in this cohort. Additional variants identified by extended panel testing suggest a heterogeneous genetic background, with occasional overlap between classical and nonclassical autoinflammatory genes. As summarized in Table 1, NSAIDs, colchicine, and systemic corticosteroids were widely used, and steroid dependency was observed in nine of 22 patients (41%). Overall, 16 patients (62%) required treatment with anakinra during the disease course and canakinumab was initiated in seven patients; however, five were switched back to anakinra due to persistent disease activity, while two patients remained on canakinumab at the last follow-up. During follow-up, cardiac tamponade was observed in one patient and was promptly treated with complete clinical recovery. Pericardiocentesis was performed in 11 patients. Comprehensive pericardial fluid analyses, including microbiological cultures, tuberculosis screening, serological tests, and cytological evaluation, were unremarkable in all cases. One patient additionally underwent pericardial biopsy, which was nondiagnostic. No patients developed permanent damage or long-term sequelae.

**Table 1:** Baseline clinical and laboratory characteristics of all patients and comparison of clinical characteristics according to anakinra treatment status

	All patients (*n* = 26)	Anakinra-treated (*n* = 16)	Anakinra-naïve (*n* = 10)	*p*
Male sex, *n (%)*	19 (73)	13 (81)	6 (60)	0.369
Age at diagnosis, months, *median (IQR)*	114 (48–168)	138 (66–162)	54 (20–168)	0.135
Follow-up duration, months, *median (IQR)*	72 (36–96)	138 (63–156)	51 (39–81)	0.677
Clinical features at initial presentation, *n (%)*				
Chest pain	16 (62)	11 (69)	5 (50)	0.425
Dyspnea	11 (42)	6 (39)	5 (50)	0.689
Fever	11 (42)	7 (44)	4 (40)	1.000
Abdominal pain	5 (19)	4 (25)	1 (10)	0.617
Pericardial effusion	26 (100)	16 (100)	10 (100)	
Pleural effusion	12 (46)	8 (50)	4 (40)	0.701
Pericardiocentesis required,* n (%)*	11 (42)	7 (44)	4 (40)	1.000
Pericardiectomy,* n (%)*	1 (4)	1 (6.3)	0 (0)	1.000
Genetic variant present, *n (%)*	11 (44)	9 (60)	2 (20)	0.099
Baseline AAR, *median (IQR)*	1.87 (0.6–8.0)	6.0 (1.88–9.0)	0.55 (0.38–1.06)	** < 0.001**
Baseline max CRP, *mean* ± *SD*	169 ± 88	147.17 ± 99.11	212.67 ± 33.29	0.057
Baseline max ESR, *mean* ± *SD*	57 ± 41	40 ± 40.8	-	NA^†^
Treatment/outcome,* n (%)*				
NSAID use	18 (69)	12 (75)	6 (60)	0.664
Colchicine use	24 (92)	16 (100)	8 (80)	0.138
Systemic corticosteroid use	22 (85)	15 (93.8)	7 (70)	0.264
Steroid response among corticosteroid-treated patients, *n (%)*				0.038^*^
Steroid dependency	9/22 (41)	7/9 (32)	2/9 (9)	
Steroids used only during flares	5/22 (23)	1/9 (5)	4/9 (18)	
Steroid-free disease control	8/22 (36)	7/9 (32)	1/9 (5)	
Canakinumab treatment	2 (8)	2 (13)	0 (0)	0.508
Treatment-free follow-up at the last visit, *n (%)*	7 (27)	3 (19)	4 (40)	0.369

*AAR* attack activity rate, *CRP* C-reactive protein, *ESR* erythrocyte sedimentation rate, *IQR* interquartile range, *NSAID* nonsteroidal anti-inflammatory drug, *NA* not available (data could not be calculated due to missing values)

^†^Baseline ESR could not be analyzed in the anakinra-naïve group because of incomplete data

^*^p value was calculated using the chi-square test with Monte Carlo simulation due to small sample size

### Clinical characteristics and disease course in patients treated with anti-interleukin-1 therapy

Most patients achieved sustained remission under daily treatment (*n* = 12/16, 75%), whereas relapse was common after dose spacing (*n* = 10/15, 63%). At the most recent follow-up of the anakinra-received group, 11 patients were receiving ongoing anakinra therapy, 11 were maintained on colchicine, two patients were treated with canakinumab, and three patients were followed without any anti-inflammatory or biologic therapy. Two patients were switched to canakinumab (150 mg/dose monthly subcutaneously). In one patient, canakinumab was initiated due to anaphylaxis to anakinra; during a total treatment duration of 34 months, this patient experienced two disease flares. In the second patient, canakinumab was started because of persistent flares under anakinra therapy; this patient had 12 attacks during the first 24 months, followed by a subsequent 36-month flare-free period. Except for one patient who developed anaphylaxis, no significant adverse events related to anakinra were observed. Treatment characteristics and outcomes of anakinra therapy are summarized in Table 2.

**Table 2:** Treatment response and clinical outcomes during anakinra therapy

	Value
Time from diagnosis to anakinra initiation (months), *median (IQR)*	4.5 (3.8–15.0)
Duration of anakinra therapy (months), *median (IQR)*	14 (6–23.3)
Daily anakinra dose, *mg/kg/day*	2–5 (max 100 mg)
Complete attack suppression, *n (%)*	12/16 (75)
Persistent flares under daily therapy, *n (%)*	4/16 (25)
Dose spacing attempted, *n (%)*	15/16 (94)
Relapse after dose spacing, *n (%)*	10/15 (63)
Discontinuation attempted, *n (%)*	4/16 (25)
Successful discontinuation, *n (%)*	3/4 (75)
Steroid discontinuation, *n (%)*	11 (69)
Colchicine discontinuation, *n (%)*	5 (31.3)
NSAID discontinuation, *n (%)*	16 (100)
Switch to canakinumab, *n (%)*	2 (13)
Serious adverse events, *n (%)*	1 (6)

*IQR* interquartile range, *NSAID* nonsteroidal anti-inflammatory drug

### Treatment response to anakinra: within-patient changes and group comparisons

**Fig. 1:**
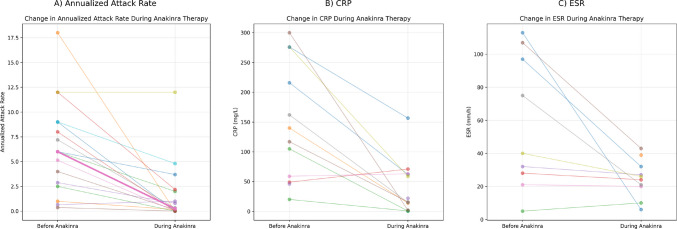
Changes in Disease Activity and Inflammatory Markers Before and During Anakinra Therapy (**A**) Annualized attack rate, (**B**) C-reactive protein, and (**C**) erythrocyte sedimentation rate before and during anakinra therapy. Each line represents an individual patient. Bold lines indicate median values

**Fig. 2:**
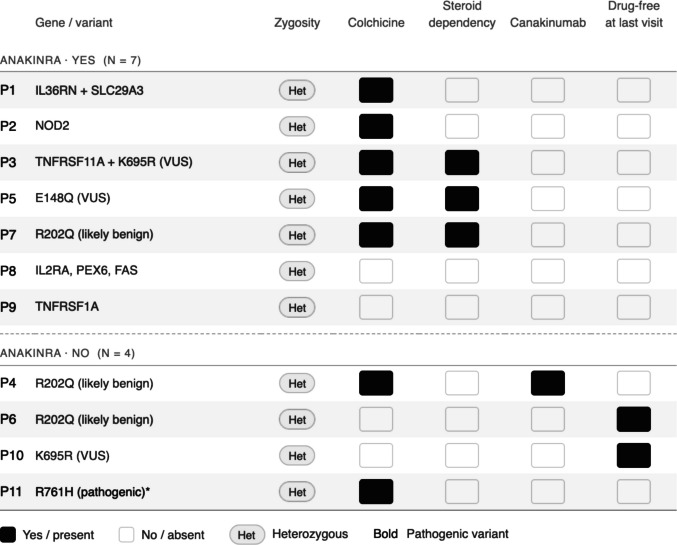
Genetic and clinical characteristics of genetically evaluated patients stratified by prior anakinra exposure. Heatmap showing genetic findings and treatment profiles of 11 patients. Each row represents one patient; columns indicate the presence (filled) or absence (empty) of clinical and treatment features. Variants were classified according to ACMG/AMP criteria, and all were heterozygous; pathogenic variants are indicated by bold gene labels. ACMG classification was applied to MEFV variants only, while variants in other genes were not formally classified due to lack of established relevance to IRP. Steroid dependency was assessed in anakinra-treated patients only, and drug-free status indicates absence of active therapy at the last follow-up. Het, heterozygous; VUS, variant of uncertain significance

Anakinra therapy was associated with marked reductions in disease activity and inflammatory burden (Table 3, Fig. 1). Significant improvements were observed in AAR and inflammatory markers, and 75% of patients achieved a ≥ 50% reduction in AAR. Patients treated with anakinra were older at diagnosis than those managed without anakinra; however, this difference was not statistically significant [median (IQR) 138 (66–162) vs 54 (20–168) months; *p* = 0.135]. Baseline demographic features, follow-up duration, and presenting clinical symptoms were comparable between groups (all *p* > 0.05). The frequency of heterozygous autoinflammatory gene variants was higher in the anakinra-treated group, but this difference was not statistically significant (60% vs 20%; *p* = 0.099). Among the 11 patients with detected genetic variants, nine required anakinra therapy at baseline, compared with seven of the 15 patients without detectable variants. Of the nine patients with genetic variants who initially received anakinra, seven remained on anakinra therapy at the last follow-up. In contrast, among the seven patients without genetic variants who were initially treated with anakinra, four continued anakinra therapy at follow-up. One patient in each group received canakinumab, while treatment-free follow-up was achieved in two and five patients in the variant-positive and variant-negative groups, respectively. Detailed genotype and treatment characteristics of individual patients are summarized in Fig. 2.

**Table 3:** Effect of anakinra on disease activity and inflammatory markers

Parameter	Before anakinra	During anakinra therapy	*p* value	Effect size
AAR, median (IQR)	6.6 (2.7–9.0)	0.25 (0–2.1)	**0.001**	*r* = 0.84
Relative AAR reduction (%)	–	89.4 (56.7–100)	–	–
CRP (mg/L)	147.2 ± 99.1	37.1 ± 44.9	** < 0.05**	dz = 1.21
ESR (mm/h)	57.6 ± 40.8	24.8 ± 11.6	** < 0.05**	dz = 0.87

Effect size measures: *r* indicates effect size for nonparametric tests, and dz represents Cohen’s dz for paired samples

*AAR* attack activity rate, *CRP* C-reactive protein, *ESR* erythrocyte sedimentation rate, *IQR* interquartile range

A significant difference was observed in baseline disease activity. Patients receiving anakinra had a markedly higher AAR compared with anakinra-naïve patients [median (IQR) 6.0 (1.88–9.0) vs 0.55 (0.38–1.06); *p* < 0.001], indicating a more severe disease course prior to biologic therapy initiation. Baseline maximum CRP levels were numerically higher in patients managed without anakinra than in those who later required anakinra (212.67 ± 33.29 vs 147.17 ± 99.11 mg/L), although this difference did not reach statistical significance (*p* = 0.057).

**Fig. 3:**
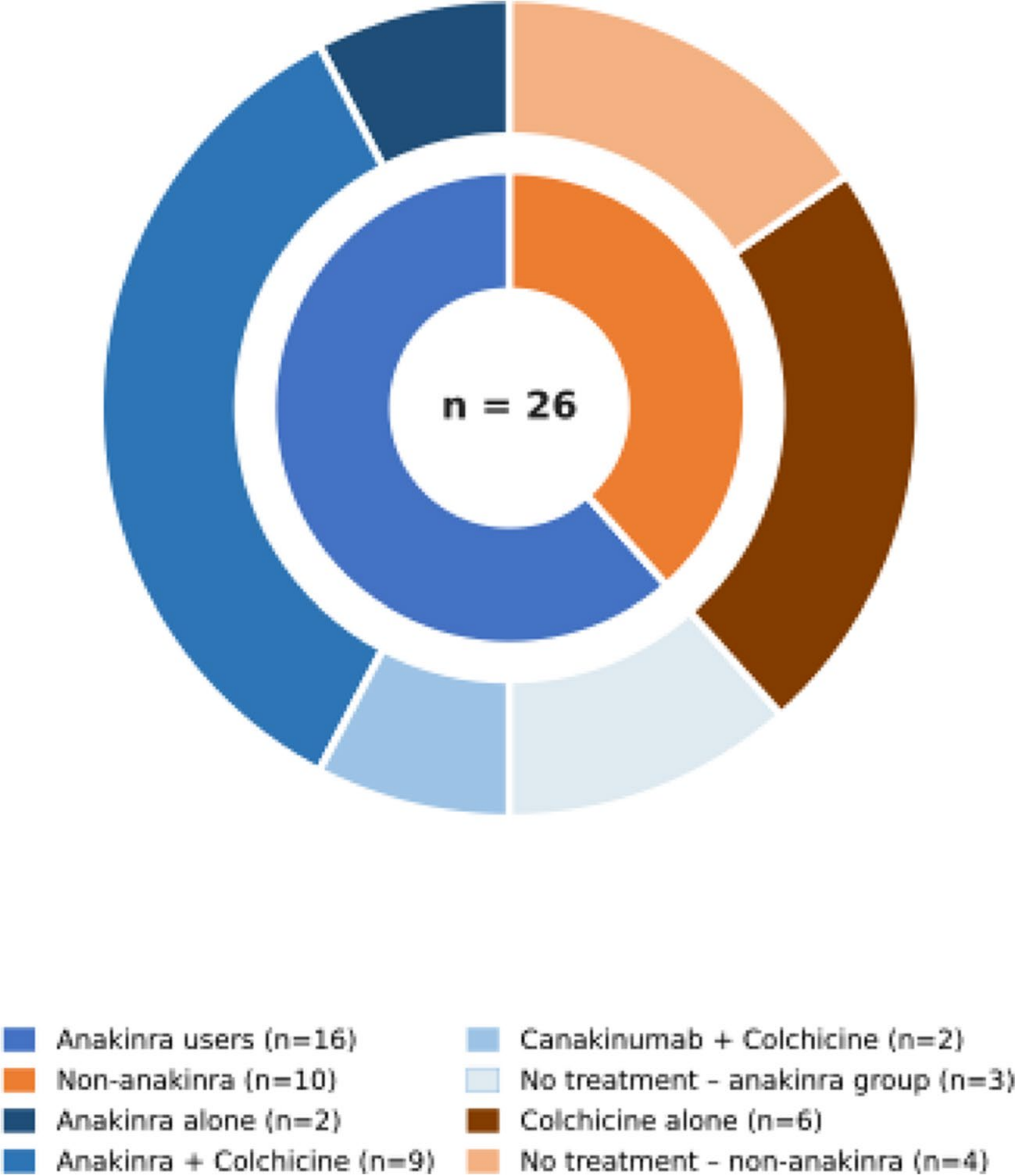
Treatment status at the last follow-up according to prior anakinra exposure. The inner ring represents patient groups based on prior anakinra exposure (anakinra users, *n* = 16; non-anakinra, *n* = 10). The outer ring shows the distribution of treatment regimens at the last follow-up within each group, including anakinra alone, anakinra plus colchicine, canakinumab plus colchicine, colchicine alone, and no treatment. Total cohort size was 26 patients

Treatment patterns, including NSAIDs, colchicine, and corticosteroids, did not differ significantly between groups. However, corticosteroid response differed significantly (*p* = 0.038), with a higher rate of steroid dependency among patients requiring anakinra, suggesting greater treatment resistance. At the last follow-up, rates of treatment-free disease control were similar between groups (*p* = 0.369). At the latest follow-up (*n* = 26), half of the patients were receiving anti-IL-1 therapy, whereas the remaining patients were maintained on colchicine alone or followed without treatment. Stratified analyses according to prior anakinra exposure showed that patients previously treated with anakinra were more likely to require ongoing biologic or anti-inflammatory therapy, whereas those without prior anakinra exposure were predominantly managed conservatively (Fig. 3).

## Discussion

Our findings strongly support an autoinflammatory-driven disease model in pediatric IRP, reflected by the high prevalence of MEFV-associated variants, frequent corticosteroid dependency, and the substantial need for IL-1 inhibition. Anakinra-treated patients represent a more severe disease subset with higher inflammatory burden and a refractory course, yet show a marked and clinically meaningful response to IL-1 blockade. Importantly, sustained disease control appears to depend on continuous therapy, as dose spacing is frequently associated with relapse. Together, these results highlight the central role of IL-1-mediated inflammation in disease pathogenesis and position anakinra as a key therapeutic strategy in severe pediatric IRP.

In this study, we evaluated 26 children with IRP and observed a male predominance, with most patients being older than 9 years at presentation, findings that are consistent with previous pediatric reports. Chest pain was the most common presenting symptom, whereas dyspnea predominated in younger children, likely reflecting age-related limitations in symptom reporting [15, 16]. Pericardial effusion was present in all patients, and pleural effusion accompanied pericardial involvement in approximately half of cases. However, as previously reported, the absence of pericardial or pleural effusion does not exclude the diagnosis of recurrent pericarditis, underscoring the heterogeneous clinical spectrum of the disease and the need for comprehensive clinical assessment beyond imaging findings alone [3, 16].

In line with previous pediatric and adult cohorts, NSAIDs, colchicine, and corticosteroids were commonly used first-line therapies in our study. However, steroid dependency occurred in a substantial proportion (9/22, 41%) of patients, consistent with reports describing frequent relapses during corticosteroid tapering [17–20]. These observations underscore the limited ability of steroid-based regimens to achieve sustained remission and support the need for effective steroid-sparing therapeutic strategies in recurrent pericarditis [21, 22].

Among anti-interleukin-1 agents, anakinra was the most frequently used therapy in our cohort, reflecting its role as a preferred steroid-sparing option in recurrent pericarditis. Sustained disease control under daily anakinra therapy in most patients is consistent with prior reports by Imazio et al., who demonstrated significant reductions in recurrence rates, healthcare utilization, and corticosteroid exposure in refractory disease [23]. Importantly, relapse frequently occurred during anakinra tapering or discontinuation, suggesting that ongoing IL-1 blockade may be necessary to sustain remission in a substantial proportion of pediatric patients, consistent with the relapsing disease course reported in previous studies [24, 25].

Canakinumab was used in a limited number of patients, with persistent disease activity observed during the initial treatment period, followed by a reduction in flare frequency over longer follow-up. Although based on small numbers, this observation parallels prior reports suggesting that alternative IL-1 inhibitors may be effective in selected cases, particularly when long-term disease control is required [22, 26, 27].

In our cohort, 75% of patients achieved a ≥ 50% reduction in annualized attack rate, with a median relative reduction approaching 90%, indicating profound suppression of disease recurrence. This response is consistent with prior studies showing that IL-1 blockade markedly reduces flare frequency, particularly in patients with frequent-relapsing or steroid-dependent recurrent pericarditis [16, 21, 23]. This marked reduction in attack burden was accompanied by large within-patient effects on CRP and ESR, indicating robust suppression of systemic inflammation. Similar concordant clinical and inflammatory responses have been reported by Finetti et al., supporting the view that IL-1 inhibition exerts a biologically coherent, disease-modifying effect in recurrent pericarditis rather than providing symptomatic relief alone [24].

Patients requiring anakinra had a higher baseline annualized attack rate, indicating a more active disease course. Autoinflammatory gene variants were observed in both groups, and although a higher proportion of patients with detected variants required anakinra therapy, this difference was not statistically significant, likely due to the limited sample size. Nevertheless, this pattern is consistent with previous reports suggesting that patients with autoinflammatory genetic variants may have a more severe or refractory phenotype and may derive particular benefit from IL-1-targeted therapies, supporting the biological plausibility of our findings [28–30].

This study has several limitations. First, its retrospective design and single-center setting may limit the generalizability of the findings. Second, the relatively small sample size reduced statistical power, particularly for subgroup analyses, including comparisons based on genetic findings. Third, treatment decisions, including initiation and tapering of anakinra, were made according to clinical judgment rather than a standardized protocol, which may have introduced variability in management. Finally, autoinflammatory gene panel testing was not systematically performed in all patients, reflecting real-world limitations in access and availability. In the tested subgroup, both MEFV gene analysis and broader autoinflammatory gene panel testing revealed variants with diverse classifications, including pathogenic, likely pathogenic, variants of uncertain significance, and likely benign variants. However, the absence of functional validation limited definitive genotype–phenotype correlations. Despite these limitations, the consistent clinical and inflammatory responses observed provide meaningful real-world insights and support the role of IL-1 blockade in pediatric IRP.

In conclusion, idiopathic recurrent pericarditis in children represents a heterogeneous and frequently relapsing disease, in which conventional therapies may be insufficient in a substantial proportion of patients. In our cohort, IL-1 blockade, particularly with anakinra, was associated with meaningful reductions in disease activity, inflammatory burden, and corticosteroid exposure, supporting its role as a steroid-sparing option in selected pediatric patients. These findings are consistent with the emerging view of recurrent pericarditis as an autoinflammatory condition and underscore the need for individualized treatment strategies. Larger, prospective studies are needed to further define optimal patient selection and long-term outcomes.

## Data Availability

All data generated or analyzed during this study are included in this published article.

## Abbreviations

AAR: Annualized attack rate
CACP: Camptodactyly–arthropathy–coxa vara–pericarditis syndrome
CRP: C-reactive protein
ESR: Erythrocyte sedimentation rate
ESC: European Society of Cardiology
FMF: Familial Mediterranean fever
IL-1: Interleukin-1
IQR: Interquartile range
IRP: Idiopathic recurrent pericarditis
NSAIDs: Nonsteroidal anti-inflammatory drugs
TRAPS: Tumor necrosis factor receptor–associated periodic syndrome

## References

[1] Tombetti E, Giani T, Brucato A, Cimaz R (2019) Recurrent pericarditis in children and adolescents. Front Pediatr 7:419 31681717 10.3389/fped.2019.00419 PMC6813188

[2] Imazio M, Lazaros G, Brucato A, Gaita F (2016) Recurrent pericarditis: new and emerging therapeutic options. Nat Rev Cardiol 13(2):99–105 26259934 10.1038/nrcardio.2015.115

[3] Adler Y, Charron P, Imazio M, Badano L, Barón-Esquivias G, Bogaert J et al (2015) 2015 ESC guidelines for the diagnosis and management of pericardial diseases. Polish Heart J (Kardiologia Polska) 73(11):1028–1091 10.5603/KP.2015.0228 26726821

[4] Alsabri M, Elsnhory AB, Ali MA, Elsayed SM, Rifai M, Elshanbary AA et al (2025) Clinical presentation, diagnosis, and outcomes of pediatric pericarditis in acute care settings: a systematic review and meta-analysis. Curr Emerg Hosp Med Rep 13(1):4

[5] Tombetti E, Mulè A, Tamanini S, Matteucci L, Negro E, Brucato A et al (2020) Novel pharmacotherapies for recurrent pericarditis: current options in 2020. Curr Cardiol Rep 22(8):59 32562029 10.1007/s11886-020-01308-y PMC7303578

[6] Cantarini L, Imazio M, Brizi MG, Lucherini OM, Brucato A, Cimaz R et al (2013) Role of autoimmunity and autoinflammation in the pathogenesis of idiopathic recurrent pericarditis. Clin Rev Allergy Immunol 44(1):6–13 21170606 10.1007/s12016-010-8219-x

[7] Cantarini L, Lopalco G, Selmi C, Napodano S, De Rosa G, Caso F et al (2015) Autoimmunity and autoinflammation as the yin and yang of idiopathic recurrent acute pericarditis. Autoimmun Rev 14(2):90–97 25308531 10.1016/j.autrev.2014.10.005

[8] Brucato A, Emmi G, Cantarini L, Di Lenarda A, Gattorno M, Lopalco G et al (2018) Management of idiopathic recurrent pericarditis in adults and in children: a role for IL-1 receptor antagonism. Intern Emerg Med 13(4):475–489 29633070 10.1007/s11739-018-1842-x

[9] Lopalco G, Rigante D, Cantarini L, Imazio M, Lopalco A, Emmi G et al (2021) The autoinflammatory side of recurrent pericarditis: enlightening the pathogenesis for a more rational treatment. Trends Cardiovasc Med 31(5):265–274 32376492 10.1016/j.tcm.2020.04.006

[10] Wang TKM, Klein AL, Cremer PC, Imazio M, Kohnstamm S, Luis SA et al (2025) 2025 concise clinical guidance: an ACC expert consensus statement on the diagnosis and management of pericarditis: a report of the American College of Cardiology Solution Set Oversight Committee. J Am Coll Cardiol 86(25):2691–2719 40767817 10.1016/j.jacc.2025.05.023

[11] Yilmaz S, Uludağ Alkaya D, Kasapçopur Ö, Barut K, Akdemir ES, Celen C et al (2018) Genotype–phenotype investigation of 35 patients from 11 unrelated families with camptodactyly–arthropathy–coxa vara–pericarditis (CACP) syndrome. Mol Genet Genomic Med 6(2):230–248 29397575 10.1002/mgg3.364 PMC5902402

[12] Ekinci RMK, Konte EK, Akay N, Gul U (2024) Familial Mediterranean fever in childhood. Turk Arch Pediatr 59(6):527 39540697 10.5152/TurkArchPediatr.2024.24188 PMC11562618

[13] Schulz-Menger J, Collini V, Gröschel J, Adler Y, Brucato A, Christian V et al (2025) 2025 ESC guidelines for the management of myocarditis and pericarditis: developed by the task force for the management of myocarditis and pericarditis of the European Society of Cardiology (ESC) Endorsed by the Association for European Paediatric and Congenital Cardiology (AEPC) and the European Association for Cardio-Thoracic Surgery (EACTS). Eur Heart J 46(40):3952–4041 40878297 10.1093/eurheartj/ehaf192

[14] Brucato A, Imazio M, Gattorno M, Lazaros G, Maestroni S, Carraro M et al (2016) Effect of anakinra on recurrent pericarditis among patients with colchicine resistance and corticosteroid dependence: the AIRTRIP randomized clinical trial. JAMA 316(18):1906–1912 27825009 10.1001/jama.2016.15826

[15] Raatikka M, Pelkonen PM, Karjalainen J, Jokinen EV (2003) Recurrent pericarditis in children and adolescents: report of 15 cases. J Am Coll Cardiol 42(4):759–764 12932616 10.1016/s0735-1097(03)00778-2

[16] Imazio M, Brucato A, Pluymaekers N, Breda L, Calabri G, Cantarini L et al (2016) Recurrent pericarditis in children and adolescents: a multicentre cohort study. J Cardiovasc Med 17(9):707–712 10.2459/JCM.0000000000000300 27467459

[17] Caorsi R, Insalaco A, Longo C, Martini G, Cattalini M, Consolini R et al (2019) SAT0490 IL-1 blockade in pediatric recurrent pericarditis: a multicentric retrospective study on the Italian cohort. Ann Rheum Dis 78:1334

[18] Imazio M, Bobbio M, Cecchi E, Demarie D, Pomari F, Moratti M et al (2005) Colchicine as first-choice therapy for recurrent pericarditis: results of the CORE (COlchicine for REcurrent pericarditis) trial. Arch Intern Med 165(17):1987–1991 16186468 10.1001/archinte.165.17.1987

[19] Imazio M, Brucato A, Cumetti D, Brambilla G, Demichelis B, Ferro S et al (2008) Corticosteroids for recurrent pericarditis: high versus low doses: a nonrandomized observation. Circulation 118(6):667–671 18645054 10.1161/CIRCULATIONAHA.107.761064

[20] Cremer PC, Kumar A, Kontzias A, Tan CD, Rodriguez ER, Imazio M et al (2016) Complicated pericarditis: understanding risk factors and pathophysiology to inform imaging and treatment. J Am Coll Cardiol 68(21):2311–2328 27884251 10.1016/j.jacc.2016.07.785

[21] Caorsi R, Insalaco A, Bovis F, Martini G, Cattalini M, Chinali M et al (2023) Pediatric recurrent pericarditis: appropriateness of the standard of care and response to IL-1 blockade. J Pediatr 256(18–26):e8 10.1016/j.jpeds.2022.11.034 36470465

[22] Lazaros G, Antonatou K, Vassilopoulos D (2017) The therapeutic role of interleukin-1 inhibition in idiopathic recurrent pericarditis: current evidence and future challenges. Front Med 4:78 10.3389/fmed.2017.00078 PMC5466978 28660191

[23] Imazio M, Andreis A, De Ferrari GM, Cremer PC, Mardigyan V, Maestroni S et al (2020) Anakinra for corticosteroid-dependent and colchicine-resistant pericarditis: the IRAP (International Registry of Anakinra for Pericarditis) study. Eur J Prev Cardiol 27(9):956–964 31610707 10.1177/2047487319879534

[24] Finetti M, Insalaco A, Cantarini L, Meini A, Breda L, Alessio M, D’Alessandro M, Picco P, Martini A, Gattorno M (2014) Long-term efficacy of interleukin-1 receptor antagonist (Anakinra) in corticosteroid-dependent and colchicine-resistant recurrent pericarditis. J Pediatr 164(6):1425–31 24630353 10.1016/j.jpeds.2014.01.065

[25] Picco P, Brisca G, Traverso F, Loy A, Gattorno M, Martini A (2009) Successful treatment of idiopathic recurrent pericarditis in children with interleukin-1β receptor antagonist (anakinra): an unrecognized autoinflammatory disease? Arthritis Rheum 60(1):264–268 19116906 10.1002/art.24174

[26] Epçaçan S, Sahin S, Kasapcopur O (2019) Anaphylactic reaction to anakinra in a child with steroid-dependent idiopathic recurrent pericarditis and successful management with canakinumab. Cardiol Young 29(4):549–551 30931868 10.1017/S1047951119000672

[27] Kilic Konte E, Akay N, Gul U, Ucak K, Derelioglu EI, Gurleyik D et al (2025) Long-term safety profile and secondary effectiveness of canakinumab in pediatric rheumatic diseases: a single-center experience. Expert Opin Drug Saf 24(8):915–923 39069814 10.1080/14740338.2024.2386370

[28] Cantarini L, Lucherini O, Cimaz R, Galeazzi M (2010) Recurrent pericarditis caused by a rare mutation in the TNFRSF1A gene and with excellent response to anakinra treatment. Clin Exp Rheumatol 28(5):802 21029567

[29] Cantarini L, Lucherini OM, Brucato A, Barone L, Cumetti D, Iacoponi F et al (2012) Clues to detect tumor necrosis factor receptor-associated periodic syndrome (TRAPS) among patients with idiopathic recurrent acute pericarditis: results of a multicentre study. Clin Res Cardiol 101(7):525–531 22311714 10.1007/s00392-012-0422-8

[30] Cantarini L, Rigante D, Merlini G, Vitale A, Caso F, Lucherini OM et al (2014) The expanding spectrum of low-penetrance TNFRSF1A gene variants in adults presenting with recurrent inflammatory attacks: clinical manifestations and long-term follow-up. Semin Arthritis Rheum 43(6):818–823 24393624 10.1016/j.semarthrit.2013.12.002

